# Investigation of Laser Ablation Quality Based upon Entropy Analysis of Data Science

**DOI:** 10.3390/e26110909

**Published:** 2024-10-27

**Authors:** Chien-Chung Tsai, Tung-Hon Yiu

**Affiliations:** Department of Semiconductor and Electro-Optical Technology, Minghsin University of Science and Technology, Hsinchu 30401, Taiwan; tonyyiu30@gmail.com

**Keywords:** laser ablation, information entropy, data science, ablation quality, material removal

## Abstract

Laser ablation is a vital material removal technique, but current methods lack a data-driven approach to assess quality. This study proposes a novel method, employing information entropy, a concept from data science, to evaluate laser ablation quality. By analyzing the randomness associated with the ablation process through the distribution of a probability value (*r_eb_*), we quantify the uncertainty (entropy) of the ablation. Our research reveals that higher energy levels lead to lower entropy, signifying a more controlled and predictable ablation process. Furthermore, using an interval time closer to the baseline value improves the ablation consistency. Additionally, the analysis suggests that the energy level has a stronger correlation with entropy than the baseline interval time (bit). The entropy decreased by 6.32 from 12.94 at 0.258 mJ to 6.62 at 0.378 mJ, while the change due to the bit was only 2.12 (from 10.84 at bit/2 to 8.72 at bit). This indicates that energy is a more dominant factor for predicting ablation quality. Overall, this work demonstrates the feasibility of information entropy analysis for evaluating laser ablation, paving the way for optimizing laser parameters and achieving a more precise material removal process.

## 1. Introduction

Entropy, a concept deeply rooted in thermodynamics [1], has long been a cornerstone in understanding the behavior of physical systems. Initially introduced in the 19th century by Rudolf Clausius and later refined by Ludwig Boltzmann, entropy represents the measure of disorder or randomness within a system. Its significance lies in its foundational role in classical thermodynamics and its broader applications across various disciplines. As we delve into the future, the concept of entropy continues to evolve, finding new realms of exploration and applications beyond its traditional domain.

In the realm of thermodynamics, entropy serves as a fundamental concept governing the direction of natural processes. It is intimately connected with the Second Law of Thermodynamics, which states that the total entropy of an isolated system always tends to increase over time. Understanding and manipulating entropy have led to significant advancements in various fields, such as heat engines, refrigeration systems, and chemical reactions. Moreover, with the advent of nanotechnology and complex systems, exploring entropy’s role in microscale phenomena promises novel insights and applications.

Modern physics has further expanded the entropy domain, intertwining it with the fabric of spacetime and the behavior of particles at the quantum level. From the pioneering works of Max Planck and Albert Einstein to the profound implications of black hole thermodynamics proposed by Stephen Hawking [2], entropy has emerged as a key concept in understanding the thermodynamic properties of black holes and the nature of information within them. The intersection of entropy with quantum mechanics and gravity opens avenues for exploring the fundamental nature of the universe and potentially unlocking the mysteries of quantum gravity.

Entropy finds a new manifestation in computer science as a crucial measure in information theory. Proposed by Claude Shannon in the mid-20th century, Shannon entropy quantifies the uncertainty or randomness in a message or data source [3,4]. It is the cornerstone for various applications in data compression, cryptography, and error detection and correction. As data science continues to burgeon, the role of entropy analysis becomes increasingly pivotal, enabling insights into data structures, pattern recognition, and anomaly detection. A recent study by Lai et al. (2024) introduced “matrix entropy” as a novel metric to evaluate large language models (LLMs) [5]. This metric builds upon information theory principles to assess how well LLMs compress data, reflecting their ability to extract relevant information and discard unnecessary elements. Future developments in this field hold promises of more sophisticated algorithms and techniques leveraging entropy for enhanced data analysis and decision-making.

In recent years, significant research efforts have focused on applying entropy concepts to laser ablation, particularly in high-entropy alloys (HEAs) [6,7,8]. HEAs are a new class of materials with unique properties, and understanding the role of entropy in their ablation behavior is crucial for optimizing their processing and performance. However, there exists a gap in exploring how information entropy, a concept from data science, can be directly applied to assess the quality of the ablation process itself. This work aims to bridge this gap by highlighting the application of information entropy in analyzing laser ablation quality through data science techniques. By analyzing the level of disorder and randomness introduced by the ablation, we aim to develop a novel approach for evaluating the effectiveness and precision of this material removal technique.

Recent advancements in the use of data science and machine learning methods have significantly advanced the evaluation and control of laser ablation processes. Grant-Jacob et al. demonstrated the potential of deep learning to predict the appearance of samples during laser ablation in real time [9,10]. Xie et al. utilized data augmentation techniques to significantly reduce the quantity of laser images required for training neural networks [11]. Zhanwen et al. employed deep learning to extract multiple features, achieving efficient laser quality assessment [12]. Moros et al. demonstrated the use of laser-induced breakdown spectroscopy (LIBS) in combination with a decision tree for classifying refractory residues [13]. Muniyallappa et al. developed a computational model to predict the threshold fluence [14]. Tang et al. trained a neural network to predict processing parameters [15]. Lara-Rodriguez et al. explored the classification of optical interferometric images using machine learning [16]. This body of literature collectively underscores the growing synergy between laser technology and data science, paving the way for more precise and intelligent control of laser ablation quality.

Key studies that have explored the application of entropy across various domains are summarized in this paper. Table 1 provides an overview of their main contributions and the roles of entropy.

Shannon entropy, a widely used method for feature selection in machine learning and data science, is typically applied in a column-wise manner [21]. Applying Shannon entropy for row-wise data selection represents a novel approach to data preprocessing. This method evaluates the uncertainty of individual data instances (rows) rather than features (columns).

Data science, a cornerstone of modern technological advancements, encompasses three critical aspects: dataset acquisition, dataset processing, and dataset analysis, each playing a pivotal role in extracting meaningful information from raw data.

Dataset acquisition involves collecting data, either self-built from theoretical models or experimental setups or extracted from existing databases and the web. The focus of this study is on self-built experimental data specifically tailored to explore the efficacy of laser ablation [22,23,24] in CMOS-MEMS (Complementary Metal Oxide Semiconductor-Micro Electro Mechanical Systems) [25,26,27] processing for a dual-function switch and humidity chip [28]. These data, derived directly from physical experiments, inherently contain elements of uncertainty due to the nature of experimental conditions and measurement limitations.

Dataset processing in this study goes beyond traditional methods. While common practices in handling numerical data include detecting and addressing outliers and applying filters with the aid of descriptive statistics, an innovative approach is introduced using Shannon entropy. This method quantifies the uncertainty inherent in each data sample, allowing for the selective removal of data points with high uncertainty. This targeted preprocessing cleanses the dataset and enhances the overall robustness of the dataset for further analysis.

Dataset analysis then builds on this refined data foundation. Employing advanced techniques such as data mining and machine learning, we analyze the processed data to extract insights and predict outcomes. In our research, removing high-uncertainty data is posited to improve the accuracy of machine learning models, particularly in predicting the outcomes of laser ablation parameters. These parameters, representing a wealth of physical information, are instrumental in understanding and optimizing the laser ablation process.

Moreover, this study illustrates the potential of integrating diverse types of information—not limited to physical, chemical, biological, or other relevant data—into artificial intelligence. This integration facilitates the development of a polymathic AI approach capable of transcending traditional boundaries and innovating solutions in CMOS-MEMS processing.

**Figure 1:**
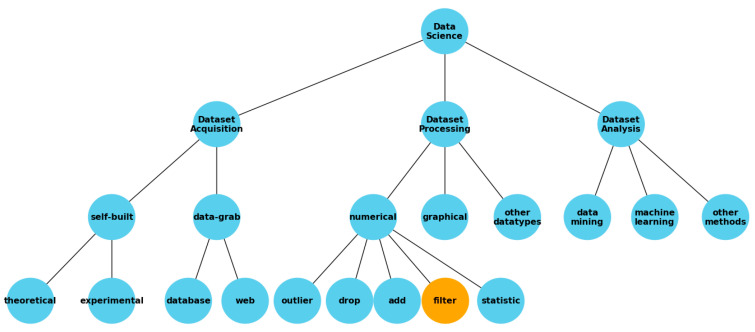
Data science workflow with emphasis on the use of Shannon entropy as a filter.

By leveraging comprehensive data science methodologies, including the use of Shannon entropy as a filter, this study aims to enhance the precision and predictability of laser ablation techniques, thereby paving the way for more sophisticated applications in semiconductor technology (Figure 1).

## 2. Materials and Methods

### 2.1. Hypothesis

The laser ablation process may involve inherent uncertainty, which can be quantified using Shannon Entropy. This quantification facilitates the identification of relevant data and physical information through data science techniques.

### 2.2. Methodology

**Figure 2:**
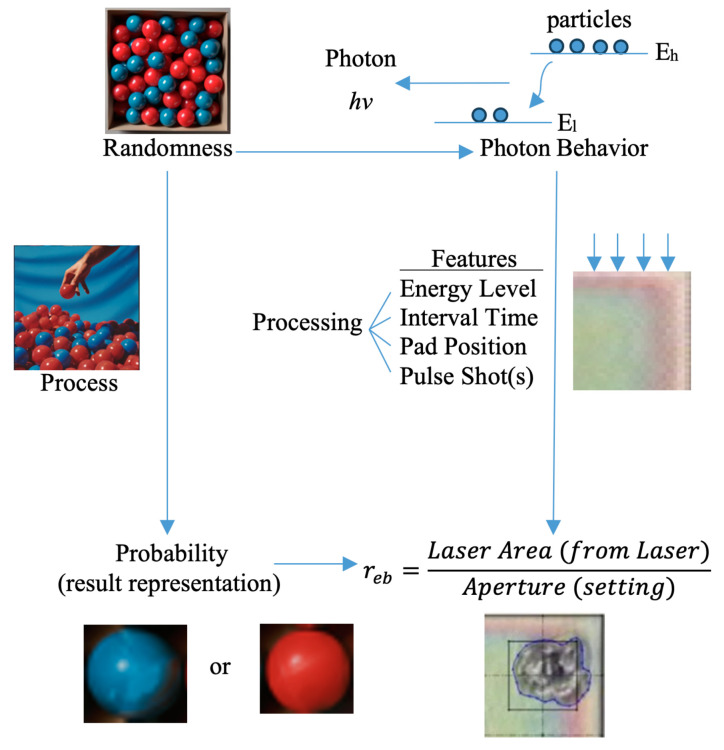
Analog between ‘randomness to probability’ and ‘photonic behavior to *r_eb_*’.

The laser aperture is the area intended to be ablated. However, the actual area ablated may not necessarily lie precisely within the aperture, and its size can also differ. To quantify the laser ablation quality, Tsai and Chan defined *r_eb_*, a metric representing the portion of the intended ablation area that undergoes ablation [29]. This study builds on their work and conducts a novel perspective of *r_eb_* by framing it as a probability, similar to how probability ranges from 0 (impossible) to 1 (certain). A higher *r_eb_* value indicates a greater ablation area within the intended aperture. Laser ablation is a photonic behavior that is a random process. As a result, the laser ablation area is also a randomness. Randomness is unpredictability in events. Probability quantifies the likelihood of outcomes. Probability theory describes and analyzes uncertainty, forming the basis for understanding and predicting random processes. This means *r_eb_* is a probability. The value is between 0 and 1. A schematic analog of how the correlation of randomness to probability and that of photonic behavior to *r_eb_* is shown in Figure 2.

### 2.3. Experimental Setup

The laser cutting system used in this study was a New Wave Research Ezlaze II device (New Wave Research, Sunnyvale, CA, USA), employing a pulsed laser ablation technique focused on material removal. The laser operates at a wavelength of 532 nm with an energy range of 0.258 mJ to 0.378 mJ. Interval times between laser shots range from 34 s to 100 s, and each sample was laser-ablated once with pulse shot(s) of 1, 3, or 5. The pad positions were either left (L) or right (R). A total of 150 samples were used, composed of poly, oxide, via, metal, and silicon nitride (Si_3_N_4_) passivation on silicon chips. This study focused on analyzing the two most dominating parameters that affect the laser ablation quality, *r_eb_*: energy and interval time.

Silicon Nitride (Si_3_N_4_) is one of the most widely used materials for passivation, and the 532 nm green laser is commonly employed for removing passivation layers. The Ezlaze system is specifically designed for removing common semiconductor materials. These choices are representative of typical CMOS-MEMS post-process scenarios. However, it is acknowledged that the findings from this study may not directly apply to other configurations, such as different materials or laser parameters.

In a laser, population inversion—where more electrons are in the excited state than the ground state—requires energy input. After a laser pulse, electrons return to the ground state, releasing photons. Energy recovery occurs during the interval between pulses, re-exciting electrons for the next shot. If this time is too short, population inversion may not occur, resulting in weaker laser pulses.

The baseline interval time (bit) [30] is a line such that the interval time is sufficient for complete energy recovery but is not optimized. The bit for energy levels ranging from 0.258 mJ to 0.378 mJ in steps of 0.03 mJ was paired with interval times ranging from 68 s to 100 s in steps of 8 s. Our investigation began by setting longer intervals as the bit and adjusting the interval times accordingly. Determining the bit is crucial for optimizing laser ablation efficiency because it helps establish a balance between process speed and ablation quality. The aim for setting the scaling factor is to seek an optimum that has a specific *r_eb_*.

The representation of the baseline interval time groups (bit groups) is as follows:

$$bit\times \frac{1}{2^{\frac{k}{2n}}}$$

where *n* = 2 and 0 ≤ *k* ≤ 4. The interval times corresponding to each energy level are listed in Table 2.

**Figure 3:**
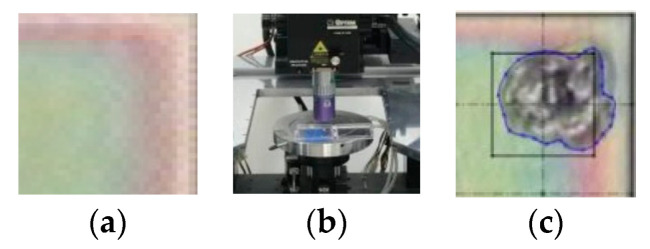
(**a**) Pad before ablation; (**b**) laser cutting system; (**c**) pad after ablation.

Figure 3a depicts the Si_3_N_4_ passivation of a silicon chip. The chip underwent laser ablation using the New Wave Research Ezlaze II device [31] at a wavelength of 532 nm, as illustrated in Figure 3b. The resulting processing outcome is shown in Figure 3c, highlighting both the square laser aperture (28.6 × 28.6 μm^2^) and the actual ablated area.

Subsequently, microscopic images of the processed chips were taken and imported to a 3D digital platform where the ablation area was delineated. The ablation area inside the aperture was divided by the area of the square aperture to obtain the laser ablation quality *r_eb_*, as shown in Figure 3c. This study focused exclusively on the areal measurement of the ablation, neglecting variations in crater depth.

Since this study reinterprets *r_eb_* as a probability within the designated area, Shannon entropy becomes a powerful tool for analysis. Shannon entropy measures the uncertainty associated with a random variable. In this case, the variable is the value of *r_eb_*, and by calculating the entropy, the level of uncertainty of the laser ablation quality can be quantified.

Shannon entropy measures the uncertainty or unpredictability of information in a random variable, quantifying the average amount of information needed to describe its possible outcomes.

The representation of Shannon entropy is as follows:

(1) $$H\left(p\right)=-\sum_{i}p_{i}{log}_{2}\left(p_{i}\right)$$

here, *p* is replaced with *r_eb_*,

(2) $$H\left(r_{eb}\right)=-\sum_{i}{r_{eb}}_{i}{log}_{2}\left({r_{eb}}_{i}\right)$$

Entropy is used to quantify the uncertainty in a statistical value based on probability theory. A high entropy would indicate significant variation in *r_eb_* values. At an intended ablation area, there are significant variations in the real area of laser ablation. Conversely, a low entropy would suggest a more uniform distribution of *r_eb_* values, signifying a more consistent ablation area. Analyzing the Shannon entropy based on the *r_eb_* distribution shows valuable insight into the consistency of the ablation process, allowing for the potential optimization of the laser ablation parameters to achieve more low-uncertainty results.

**Figure 4:**
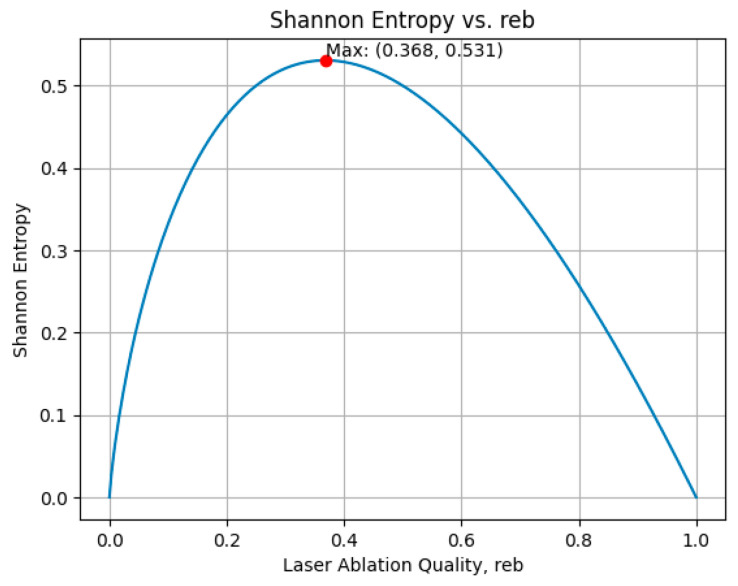
The entropy of one example varied with *r_eb_*.

In applying the entropy formula detailed in Equation (2), the relationship between Shannon entropy and laser ablation quality (*r_eb_*) was calculated for a single example (*i* = 1). Figure 4 illustrates this relationship through a plotted curve that peaks at *r_eb_* = 0.368, indicating the maximum entropy value obtained for one example. Since 150 examples (*i* = 150) were adopted in this study, the negative sum of *r_eb_* times the logarithm base 2 of *r_eb_* was calculated for all 150 examples.

If *r_eb_* surpasses 0.368, higher *r_eb_* leads to lower entropy, resulting in low uncertainty. A lower *r_eb_* leads to higher entropy, resulting in high uncertainty.

## 3. Results

### 3.1. The Effect of Energy Level

**Figure 5:**
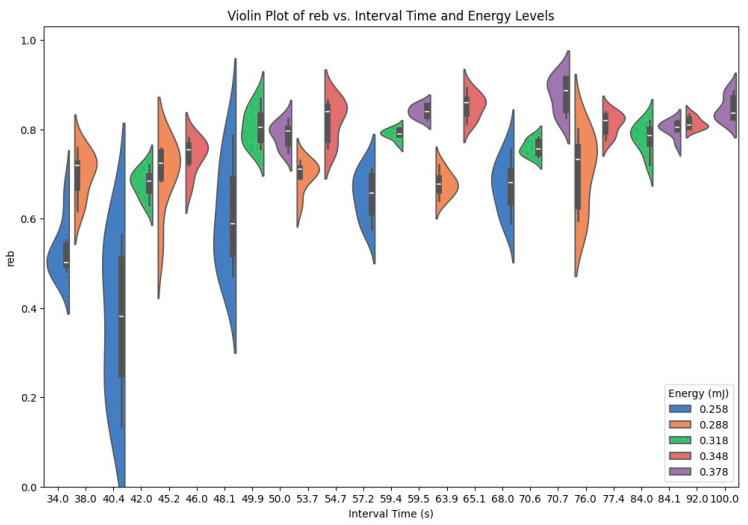
Violin plot of *r_eb_* varying with energy and interval time.

The violin plot in Figure 5 depicts the distribution of *r_eb_* (ranging from 0.13 to 0.92) across different energy levels (which range from 0.258 millijoules to 0.378 millijoules in steps of 0.03) and interval times (which range from 34 s to 100 s).

The violins show the spread of the *r_eb_* values for each combination of energy level and interval time. The white line within each violin represents the median *r_eb_* value, the thick black bar represents the interquartile range (IQR), and the thinner lines (whiskers) extend to the rest of the distribution.

The 25 tick labels on the x-axis in Figure 5 reflect the bit groups for each energy level. The rightmost violin of each energy level (or each color) corresponds to *k* = 0 (bit group). Every violin to the left increases the *k* value by 1. For instance, for the 0.258 mJ blue violins, the rightmost violin at 68 s belongs to the bit group (*k* = 0), the next left violin at 57.2 s belongs to the bit/2^(1/4) group (*k* = 1), the next left violin at 48.1 s belongs to the bit/2^(1/2) group (*k* = 2), the next left violin at 40.4 s belongs to the bit/2^(3/4) group (*k* = 3), and the leftmost violin at 34 s belongs to the bit/2 group (*k* = 4).

**Figure 6:**
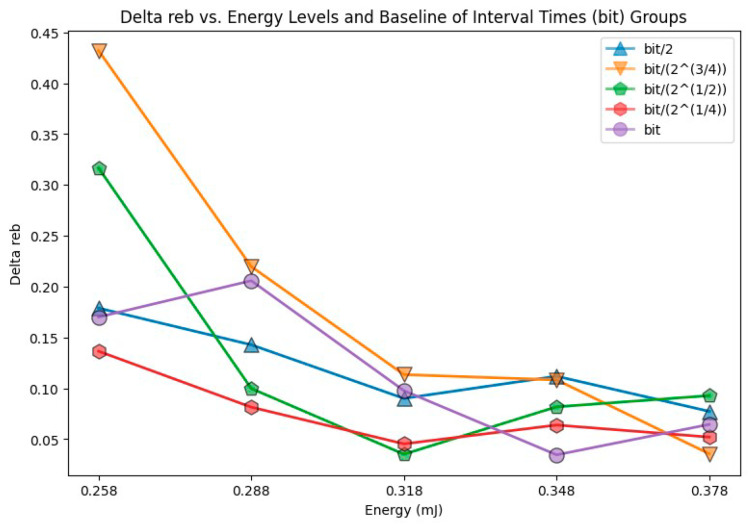
Δ*r_eb_* varying with energy and interval time.

Figure 6 focuses on the delta *r_eb_* (denoted by Δ*r_eb_*, the difference between max *r_eb_* and min *r_eb_*) for each of the 25 violins in Figure 5 (5 energy levels × 5 interval times per level). Figure 6 discards the rest of the data distribution information from Figure 5 (such as median and quartiles).

**Figure 7:**
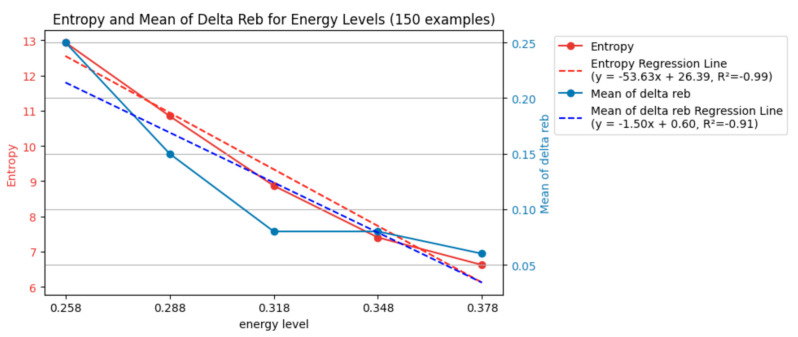
Entropy and mean of Δ*r_eb_* of each energy level (150 examples).

The mean of these 25 Δ*r_eb_* values was calculated according to their energy level. Since there were five energy levels, this process resulted in five mean Δ*r_eb_* values, as shown in Figure 7.

To quantify the uncertainty of the data in the violin plot (150 points), the entropy and mean of Δ*r_eb_* were computed.

There were five unique energy levels, with 30 data points each. The entropy for each data point was computed and then summed according to their energy level with Equation (2), as shown in Figure 7. By taking the mean of the variation in *r_eb_* (Δ*r_eb_*) across different interval times (from bit/2 through bit) for each energy, the variation in *r_eb_* (Δ*r_eb_*) could be compared with various energy levels. This approach transformed Figure 6 to the blue line of Figure 7, which simplified the data representation and enabled an analysis of how energy impacts Δ*r_eb_*.

Figure 7 quantifies the relationship between the energy level and uncertainty in the ablation process. It presents two key metrics: entropy and mean Δ*r_eb_*. Based on the regression lines obtained using scipy.stats.linregress (SciPy version 1.13.1), both metrics decreased as the energy level increased. For example, at the lowest energy level (0.258 mJ), the entropy was 12.94, indicating high uncertainty. However, at the highest energy level (0.378 mJ), the entropy dropped to 6.62, signifying a more predictable ablation process.

This trend is further corroborated by the mean Δ*r_eb_* values. The figure shows that the mean Δ*r_eb_* progressively decreased with increasing the energy level. For instance, the mean Δ*r_eb_* was 0.25 at the lowest energy level, whereas at the highest, it reduced to 0.06. This decrease suggests a more specific distribution of *r_eb_* values, indicating a more consistent ablation area across different laser pulses.

Figure 7 shows that when the entropy was low, its uncertainty was also low, and its variation (Δ*r_eb_*) remained small and more specific. When the entropy was high, the variation broadened.

In essence, the decrease in entropy and the decrease in mean Δ*r_eb_* with increasing energy levels point toward a more controlled and predictable laser ablation process. By delivering more energy, the laser interacted more consistently with the material, resulting in less variation and lower uncertainty in the final ablation area.

### 3.2. The Effect of Baseline of Interval Time (Bit)

**Figure 8:**
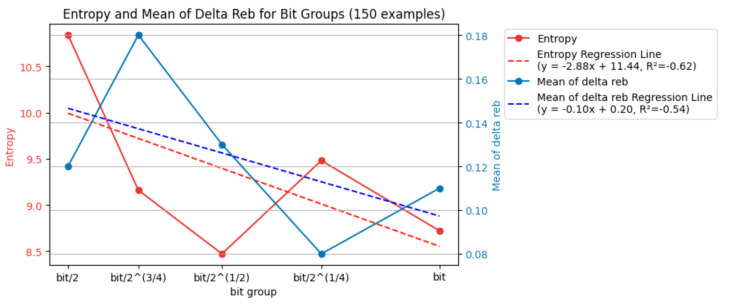
Entropy and mean of Δ*r_eb_* of each bit group (150 examples).

Similar to Figure 7, Figure 8 quantifies the uncertainty in the laser ablation process. However, Figure 7 focuses on the entropy and mean Δ*r_eb_* for each energy level. Figure 8, on the other hand, calculates these values for each bit group.

The data in Figure 8 support the idea that a more consistent interval time between laser pulses (closer to the baseline bit value) can lead to a more controlled and predictable laser ablation process.

The entropy values varied across the different bit groups but showed a generally decreasing trend as the bit group approached the baseline bit value (bit). For example, the entropy was highest at bit/2 (10.84) and lowest at bit (8.72). This suggests that using an interval time closer to the baseline (bit) reduces the uncertainty in the ablation process.

The mean Δ*r_eb_* values also decreased as the bit group approached the bit value. The highest value (0.18) was at bit/2^(3/4) and the lowest (0.08) was at bit/2^(1/4). A lower mean Δ*r_eb_* indicates a more specific concentration of *r_eb_* values, signifying a more consistent ablation area across different laser pulses.

Overall, the entropy and mean Δ*r_eb_* decreased as the bit group moved closer to the baseline bit value (bit). This suggests that a more consistent interval time between laser pulses leads to a more controlled and predictable ablation process.

These findings from Figure 8 complement the observations from Figure 7. Figure 7 shows that higher energy levels (delivering more energy) resulted in a lower entropy and mean Δ*r_eb_*. Figure 8 indicates that using an interval time closer to the baseline (bit) also reduced the uncertainty and increased the consistency in the ablation process. This suggests that delivering more energy and using a consistent interval time (around the baseline bit) are essential for achieving a more controlled and predictable laser ablation. 

While both the energy level and baseline interval time (bit group) influenced the uncertainty and consistency of the ablation process, the data suggest that the effect of energy might be more pronounced. Recalling Figure 7, the entropy and mean Δ*r_eb_* values consistently decreased with increasing energy levels. This indicates a more substantial and predictable reduction in uncertainty and a tighter distribution of *r_eb_* values (more consistent ablation area) with higher energy input. Figure 8 shows a trend where the entropy and mean Δ*r_eb_* generally decreased as the bit group approached the baseline bit value, but the changes were less prominent. For instance, the percentage difference in the entropy value from the highest at bit/2 (10.84) to the lowest at the bit (8.72) was −20%, whereas the decrease observed across energy levels in Figure 7 from the highest at 0.258 mJ (12.94) to the lowest at 0.378 mJ (6.62) was −49%. Similarly, the decrease in the mean Δ*r_eb_* across the bit groups was smaller than that observed across the different energy levels in Figure 7. The mean Δ*r_eb_* was highest with the bit/2 ^(3/4) group (0.18) and lowest with the bit/2^ (1/4) group (0.08), resulting in a −56% difference, whereas the mean Δ*r_eb_* was highest at 0.258 mJ (0.25) and lowest at 0.378 mJ (0.06), showing a −76% change. Overall, this comparison suggests that while using a consistent interval time around the baseline can improve the ablation process, the effect of increasing the energy level on reducing uncertainty and enhancing consistency appears to be more significant.

Dividing the data into five groups based on the baseline concept, entropy corresponded to *r_eb_* better than energy. Energy corresponded to a larger effect on entropy. Our processing, from the perspective of energy, is better explained using entropy.

### 3.3. Dataset Size Optimization

This study is a step towards a selective, controlled, and predictable (with low uncertainty) removal of Si_3_N_4_ passivation atop CMOS circuitry utilizing laser ablation to foster wire bonding. In this step, we focus on leveraging entropy and the mean of the variation in *r_eb_* (Δ*r_eb_*). Section 3.2 shows that some data exhibited a lower laser ablation quality (*r_eb_*), resulting in higher entropy. Some data exhibited a considerable variation in *r_eb_*, leading to a high mean Δ*r_eb_*. To leverage the dataset to obtain high predictability with input features (mainly the operation energy and interval time), especially with specific applications for our case, it is highly rational to remove part of the data, as data with low *r_eb_* do not match the aim of the effective removal of material, and data with high uncertainty make the prediction of *r_eb_* difficult. During this process, inspecting the change in entropy and mean Δ*r_eb_* provides insights into whether the removed data are redundant. Some preliminary thoughts include removing data with low operation energy or low *r_eb_* or removing specific bit groups.

Figure 7 shows a trend from broader to more specific in terms of entropy and the mean of Δ*r_eb_*. Using “broad” and “specific” emphasizes the probabilistic concept of entropy and the mean of Δ*r_eb_*.

The objective of displaying Δ*r_eb_* is to emphasize whether *r_eb_*’s nature is specific or broad. Since it is already confirmed that low energy corresponds to high entropy and high uncertainty, resulting in broader variation in *r_eb_*, data points with 0.258 mJ were removed, leaving 120 data points. The remaining four groups should demonstrate relatively more specific characteristics. The mean of *r_eb_* for each energy level was as follows: 0.258 mJ: 0.57, 0.288 mJ: 0.69, 0.318 mJ: 0.76, 0.348 mJ: 0.81, and 0.378 mJ: 0.83. The orange violins, representing the second-lowest energy level, 0.288 mJ, displays narrower and more specific variations, with *r_eb_* consistently averaging above 0.6.

This work considers entropy and Δ*r_eb_* when reducing the dataset size. The bit/2^(3/4) group had low *r_eb_* and large boxes, and the bit/2 group had the shortest interval time. Thus, they did not yield high-quality laser ablation. New data from Figure 8 are available, where bit/2, bit/2^(3/4), and bit/2^(1/4) all exhibited relatively high entropy (information). However, a further comparison of Δ*r_eb_* highlights the specificity, with the best match being the first two groups.

**Figure 9:**
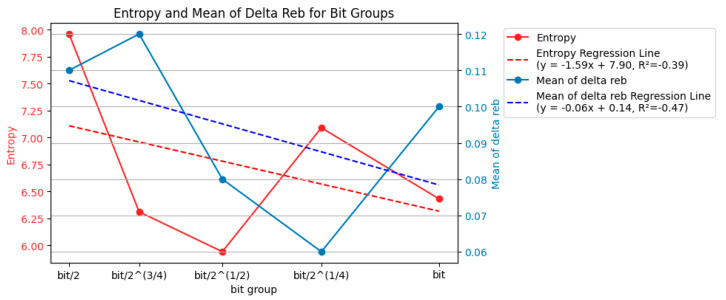
Entropy and mean of Δ*r_eb_* of each bit group (120 examples, 0.258 mJ removed).

By removing the data with 0.258 mJ and focusing the analysis on a bit as the primary axis, Figure 9 demonstrates the entropy and mean Δ*r_eb_* modifications, showing fluctuations. However, the removal of 0.258 mJ did not indicate a monotonic increase or decrease in entropy and mean Δ*r_eb_*. There was no clear trend of broadening or becoming more specific in terms of entropy and mean Δ*r_eb_* as the bit value increased. Energy changes did not affect the primary axis of analysis based on bit in terms of entropy. If there were a correlation, one meaningful adjustment (addition or removal) could lead to a better-explained correlation using the concept of entropy. From the perspective of information entropy, there was no correlation between energy and bit groups in terms of entropy. The impact of the bit itself on entropy was inconsistent and unrelated. Therefore, manipulating a correlated energy parameter would not significantly alter the analysis.

**Figure 10:**
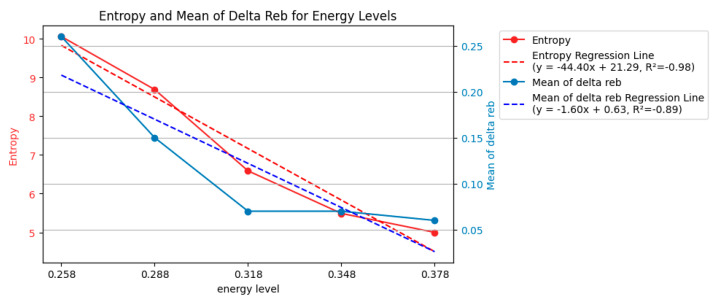
Entropy and mean of Δ*r_eb_* of each energy level (120 examples, bit/2 removed).

**Figure 11:**
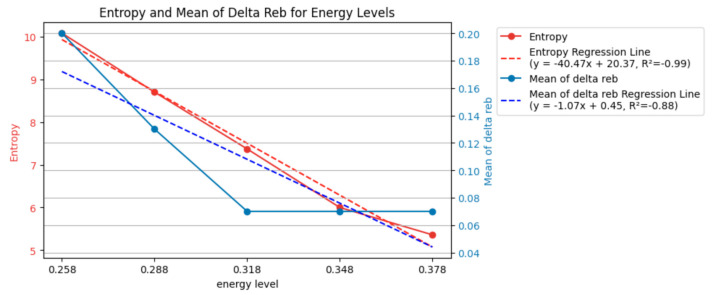
Entropy and mean of Δ*r_eb_* of each energy level (120 examples, bit/2^ (3/4) removed).

**Figure 12:**
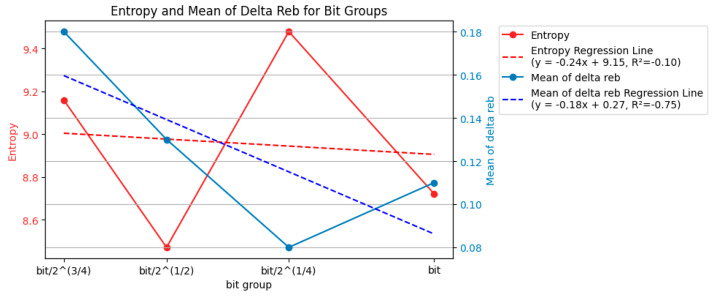
Entropy and mean of Δ*r_eb_* of each bit group (120 examples, bit/2 removed).

**Figure 13:**
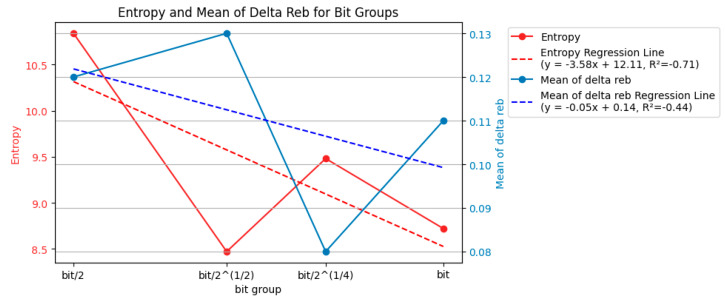
Entropy and mean of Δ*r_eb_* of each bit group (120 examples, bit/2^ (3/4) removed).

Figure 10 and Figure 11 present similar findings. Energy strongly correlated with entropy rather than bit. Figure 12 and Figure 13 fluctuate similarly to Figure 9, as evidenced by the entropy. The correlation between the concept of the baseline and entropy did not exhibit a monotonic increase or decrease; instead, it showed vibration. The impact of the bit groups based on entropy analysis was weaker than that of the energy level in terms of the uncertainty of *r_eb_*. As the parameters in this system, energy and bit behaved differently. Higher energy led to a higher probability of achieving a high *r_eb_*, resulting in low entropy and more specific *r_eb_*. However, longer bit values did not exhibit a monotonic decrease or increase in *r_eb_*, leading to fluctuating entropy and an inability to draw specific or broad conclusions.

In the future, predictions will be more accurate using energy compared to bit. Additionally, the correlation between *r_eb_* and energy will consistently be higher than that of *r_eb_* and bit.

## 4. Conclusions

This study introduces an intrinsic approach to assessing laser ablation quality through data science and entropy analysis. It defines “*r_eb_*” as the portion of the intended ablation area that undergoes ablation and reinterprets this as a probability. Shannon entropy measures uncertainty based on the distribution of *r_eb_* values. The findings suggest that higher energy levels reduce uncertainty and give a more consistent ablation area, indicating a more controlled process with increased energy delivery. At the lowest energy level (0.258 mJ), the entropy was 12.94, indicating a high level of uncertainty. However, at the highest energy level (0.378 mJ), the entropy dropped to 6.62, signifying a more predictable ablation process. Additionally, tuning the interval time closer to the baseline interval time, bit, enhanced the ablation consistency. The entropy was highest at bit/2 (10.84) and lowest at bit (8.72). Further, optimizing the dataset revealed the energy level’s stronger correlation with entropy compared to the baseline interval time. Entropy decreased by 6.32 from 12.94 at 0.258 mJ to 6.62 at 0.378 mJ, while the change due to the baseline interval time was only 2.12 (from 10.84 at bit/2 to 8.72 at bit). This study demonstrates the viability of employing entropy analysis to evaluate laser ablation quality, providing insights into optimizing laser parameters for enhanced control and predictability. Future research will focus on refining the methodology and assessing its applicability across various laser ablation applications.

## Figures and Tables

**Table 1:** Summary of key studies exploring the application of entropy in various domains.

Authors	Year	Main Contribution	Role of Entropy	Data Science Aspects	Type of Entropy
Cherednichenko et al. [17]	2024	Integrates Shannon entropy with rough set theory for model performance evaluation.	Quantifies uncertainty and information content in granules.	Analysis	Shannon entropy
Fang et al. [18]	2024	Introduces conditional entropy estimators for assessing the predictability of regression problems.	Measures uncertainty about the target variable given the feature set.	Analysis	Conditional entropy
Noroozi et al. [19]	2024	Uses symbolic transfer entropy (STE) and consensus-nested cross-validation (CN-CV) for feature selection and classification in eSports.	Measures information flow between sensors to capture player coordination and reactions.	Preprocessing	Symbolic transfer entropy
Ratnasingam et al. [20]	2023	Proposes a distance-correlation-based feature selection method for Random Forest regression.	Uses distance correlation to measure dependencies between features.	Preprocessing	Not applicable (uses distance correlation)
Juszczuk et al. [21]	2021	Introduces an entropy-based approach to estimating data difficulty for classification tasks, demonstrating the effectiveness of entropy in feature selection.	Measures data complexity and uncertainty to guide feature selection and improve classifier performance.	Preprocessing	Shannon entropy
This study	2024	Proposes a method for evaluating laser ablation quality (*r_eb_*) using Shannon entropy for data selection.	Uses Shannon entropy to quantify the uncertainty of laser ablation quality based on the distribution of *r_eb_* values.	Preprocessing	Shannon entropy

**Table 2:** Parameters of energy and interval time.

Energy (mJ)	Interval Time (s)				
	bit Group	bit/2^(1/4) Group	bit/2^(1/2) Group	bit/2^(3/4) Group	bit/2 Group
0.258	68	57.2	48.1	40.4	34
0.288	76	63.9	53.7	45.2	38
0.318	84	70.6	59.4	49.9	42
0.348	92	77.4	65.1	54.7	46
0.378	100	84.1	70.7	59.4	50

## Data Availability

Data are contained within the article.
